# Uridine and pyruvate protect T cells’ proliferative capacity from mitochondrial toxic antibiotics: a clinical pilot study

**DOI:** 10.1038/s41598-021-91559-8

**Published:** 2021-06-18

**Authors:** Stefano Battaglia, Stefania De Santis, Monica Rutigliano, Fabio Sallustio, Angela Picerno, Maria Antonia Frassanito, Ingo Schaefer, Angelo Vacca, Antonio Moschetta, Peter Seibel, Michele Battaglia, Gaetano Villani

**Affiliations:** 1 Department of Interdisciplinary Medicine, “Aldo Moro” University of Bari, 70124 Bari, Italy; 2 Department of Emergency and Organ Transplants, “Aldo Moro” University of Bari, 70124 Bari, Italy; 3 Department of Pharmacy-Drug Science, “Aldo Moro” University of Bari, 70126 Bari, Italy; 4 Department of Biomedical Sciences and Human Oncology, “Aldo Moro” University of Bari, 70124 Bari, Italy; 5 Molecular Cell Therapy, BBZ, Medical Faculty, University of Leipzig, 04103 Leipzig, Germany; 6 Department of Basic Medical Sciences, Neuroscience and Sense Organs, “Aldo Moro” University of Bari, 70124 Bari, Italy

**Keywords:** Antibiotics, Mitochondria, Urinary tract infection

## Abstract

Antibiotics that inhibit bacterial protein or nucleic acid synthesis and function can exert an off-target action on mitochondria (mitotoxic antibiotics), making actively dividing mammalian cells dependent on uridine and pyruvate supplementation. Based on this rationale, we carried out, for the first time, a randomized pilot study in 55 patients with asymptomatic bacteriuria or positive sperm culture, each treated with a single mitotoxic antibiotic with or without oral supplementation of uridine + pyruvate (Uripyr, Mitobiotix, Italy). The in vivo and ex vivo data show a a 3.4-fold higher value in the differential (before and after the antibiotic treatment) lymphocytes count and a 3.7-fold increase in the percentage of dividing T cells, respectively, in the Uripyr vs the control group. Our findings lay the groundwork to enhance the synergy between antibiotics and the immune system in order to optimize the administration protocols and widen the application potentials of antibiotic therapies as well as to re-evaluate old “forgotten” molecules to fight bacterial infections in the antibiotics resistance era.

## Introduction

Antibiotics are natural or synthetic molecules used to fight microbial infections thanks to their ability to kill bacteria (bactericidal effect) and/or to prevent their reproduction (bacteriostatic effect)^1,2^. Since approved antibiotics, at therapeutic dosages, are generally safe and well-tolerated by patients, they have been widely used in clinical practice in the last century. However, most of them have side effects ranging from fever, nausea and major allergic reactions, to variations in the blood count, liver malfunction and damage to bone marrow^2^.

Based on their mechanisms of action, antibiotics can be subdivided into three main groups: (1) inhibitors of cell wall synthesis/function (e.g. β-lactams); (2) inhibitors of nucleic acids synthesis/function (e.g. sulfonamides, quinolones); (3) inhibitors of protein synthesis (e.g. aminoglycosides, macrolides, tetracyclines, oxazolidinones, amphenicols). Antibiotic adverse effects are not fully understood at the cellular and molecular level. While antibiotics of the first group are selective for bacterial structures, those binding bacterial ribosomes or enzymes involved in DNA replication and transcription have been shown to exert off-target toxicity on mitochondria (mitotoxicity)^3^.

Mitochondria are semi-autonomous organelles possessing a replicating circular DNA (mtDNA) that encodes 13 protein subunits of the oxidative phosphorylation (OXPHOS) complexes, plus 22 transfer RNAs (tRNAs) and two ribosomal RNAs (12S and 16S rRNAs) needed for their protein synthesis machinery. Due to the endosymbiotic origin of mitochondria, organellar proteins, ribosomes, and DNA share a close evolutionary proximity with their bacterial counterparts.

Mitochondria have the main task of generating ATP through OXPHOS; hence, inhibiting mtDNA replication and/or expression will force a cellular energy metabolism shift to anaerobic glycolysis. This change is exacerbated in the phenotype of mtDNA-depleted (rho0) cells that require exogenous pyruvate and uridine to bypass the need of a functional mitochondrial respiration for their survival and proliferation^4^. Pyruvate supplementation is needed to replenish cellular NAD^+^ through lactate dehydrogenase activity^4^, as well as to support aspartate biosynthesis^5^ when the mitochondrial respiratory capacity is lacking or severely reduced. Uridine supplementation complements the lack of endogenous pyrimidine biosynthesis due to stalling of the dihydroorotate dehydrogenase (DHODH) activity which is coupled to the respiratory chain to carry out the efficient oxidation of mitochondrial ubiquinol^6^. Interestingly, it has recently been demonstrated that the fluoroquinolone antibiotic ciprofloxacin that targets bacterial DNA gyrase and topoisomerase IV, also inhibits mitochondrial topoisomerase 2, altering mtDNA topology and causing a drastic reduction of the mtDNA copy number and mtDNA transcription, with subsequent stalling of cell proliferation and differentiation in human cells^7^. Therefore, it is evident that the adverse effects of mitotoxic antibiotics may be more marked in actively dividing cells, such as skin cells, epithelial cells of the gastrointestinal tract and blood cells, in particular immune cells.

Indeed, some of the best known adverse effects like neutropenia, anaphylaxis and allergy underlie the presence of a tight interaction between antibiotic treatment and immune response. The search for immune implications of antibiotic therapies has mostly addressed lymphocytes transformation, chemotaxis, and hypersensitivity^1,8–10^. Moreover, in the context of the ever-increasing evidence of the involvement of mitochondria in the proper function of the adaptive and innate immune system^11,12^, immune dysfunctions and an increased incidence of infections have been reported in patients with mitochondrial diseases^13,14^. On the other hand, a direct effect of mitotoxic antibiotics on the respiratory capacity of immune cells has also been demonstrated in human and animal studies^15,16^.

To translate the above observations into clinical practice, we carried out a randomized pilot study in patients treated with mitotoxic antibiotics (levofloxacin, ciprofloxacin, tigecycline, doxycycline, linezolid, erythromycin), with or without oral supplementation of 450 mg of uridine and 6 g of pyruvate (Uripyr, Mitobiotix, Italy) per day. Data obtained from this study demonstrate, for the first time, that simple metabolic supplementation is able to rescue the immune-suppressive action of mitotoxic antibiotics. Our findings will be discussed in terms of their impact on different aspects of antibiotic medicine.

## Results

### In vivo effect of Uripyr supplementation on patients treated with mitotoxic antibiotics

**Figure 1:**
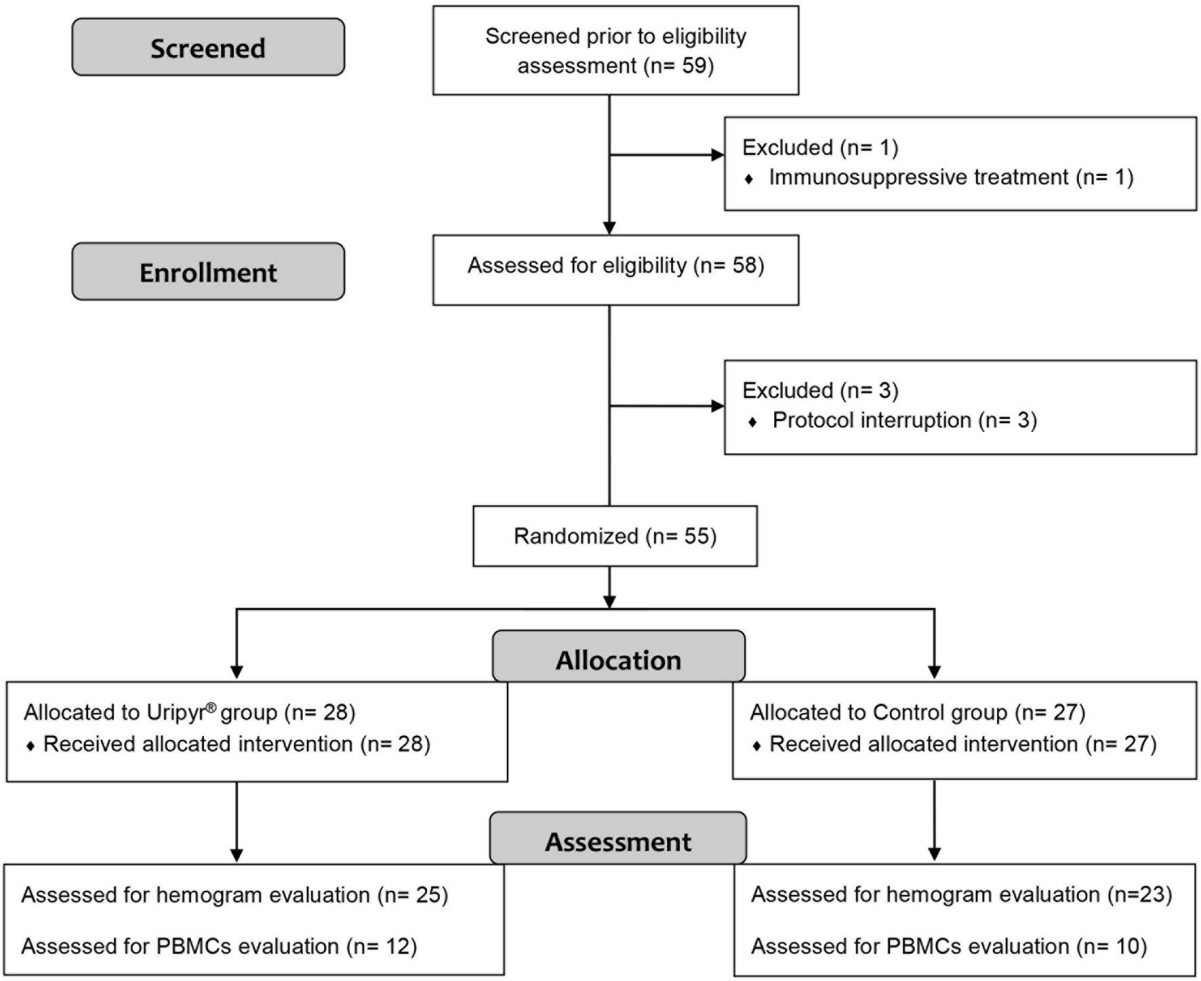
Flow chart for the selection of study population.

The subjects enrolled in this study presented asymptomatic bacteriuria (ABU) and/or positive sperm culture. According to EAU guidelines^17^, asymptomatic bacteriuria (ABU) is defined as a bacterial growth in the urine of ≥ 10^5^ CFU/ml in patients without lower urinary tract symptoms^18^. ABU is a common situation in clinical practice and can often be due to bacterial perineal colonization, particularly in elderly subjects suffering from benign prostatic hyperplasia (BPH) with residual urine. Unlike in urine culture, the presence of bacteria in sperm culture has no referential bacterial growth values. Following EAU guidelines, antibiotic treatment of ABU or positive sperm culture is not indicated to avoid the risk of selecting antimicrobial resistance or of eradicating a potentially protective strain. The indication for treatment in our selected population was the prophylaxis for invasive urological procedures breaching the mucosa, such as cystoscopy, retrograde intrarenal surgery (RIRS), prostate biopsy and other endourological maneuvers and/or as an attempt to ameliorate the quality of semen prior to the assisted reproduction^19^. The demographic and clinical features of the general population are summarized in Table 1. Data from subpopulations of patients analyzed for hemogram and PBMCs evaluations are reported in Supplementary Tables 1 and 2, respectively. In each table, the number and the relative percentage of subjects treated with different types of antibiotics are indicated (see Fig. 1, Table 1, and Supplementary Tables 1 and 2).

**Table 1:** Demographic and clinical features of the enrolled patients.

Variable	Uripyr group	Control group	NA
Number	28	27	–
Age (years)*	58.32 ± 16.12	53.78 ± 16.73	
BMI (kg/m^2^)*	25.46 ± 2.86	25.81 ± 3.30	5
WBC (T_0_) (× 10^3^/μL)**	6.50 [1.89]	6.43 [1.76]	4
WBC (T_END_) (× 10^3^/μL)**	6.31 [1.54]	5.54 [1.67]	5
Δ WBC (× 10^3^/μL)**	0.01 [1.72]	− 0.60 [1.81]	2
Neutr. count (T_0_) (× 10^3^/μL)*	4.20 ± 1.67	4.08 ± 2.00	3
Neutr. count (T_END_) (× 10^3^/μL)*	3.46 ± 0.93	3.01 ± 0.91	5
Δ Neutr. (× 10^3^/μL)*	− 0.58 ± 1.75	− 1.25 ± 2.39	2
Mono. count (T_0_) (× 10^3^/μL)*	0.50 ± 0.20	0.43 ± 0.13	4
Mono. count (T_END_) (× 10^3^/μL)*	0.46 ± 0.15	0.43 ± 0.19	5
Δ Mono. (× 10^3^/μL)*	− 0.02 ± 0.18	− 0.04 ± 0.19	2
Lymph. count (T_0_) (× 10^3^/μL)**	2.05 [0.52]	1.86 [0.77]	4
Lymph. count (T_END_) (× 10^3^/μL)**	2.36 [0.92]	1. 94 [0.94]	5
Δ Lymph. (× 10^3^/μL)**	0.36 [0.45]	0.09 [0.62]	2
**Sex**			
Male	21 (75.00)	23 (85.18)	
Female	7 (25.00)	4 (14.81)	
**Smoke**			
Non smokers	25 (89.28)	22 (84.61)	1
Smokers	3 (10.71)	4 (15.38)	
**General comorbidity**			
Absence	16 (57.14)	16 (59.25)	
Presence	12 (42.85)	11 (40.74)	
**Nephrological disease**			
Absence	26 (92.85)	24 (88.88)	
Presence	2 (7.14)	3 (11.11)	
**Nephrolithiasis**			
Absence	26 (92.85)	26 (96.29)	
Presence	2 (7.14)	1 (3.70)	
**Germ location**			
Urine	18 (64.28)	11 (40.74)	
Sperm	10 (35.72)	16 (59.26)	
**Antibiotic treatment**			
Quinolones	20 (71.42)	16 (59.25)	
Macrolides	0 (00.00)	4 (14.81)	
Tetracyclines	8 (28.57)	6 (22.22)	
Oxazolidinones	0 (00.00)	1 (3.70)	

Values are expressed as mean ± Standard Deviation or median [IQR] respectively for normal (*) and non-normal (**) distributed numeric variables, and with n (%) for categorical ones. Each item was compared among the 2 groups using t-test or Mann–Whitney’s U test for quantitative variable and Pearson χ^2^ test for categorical ones. A level of significance of P < 0.05 (two-sided) was used to compare Uripyr and control group.

*NA* number of patients for which the corresponding data were not available, *BMI* Body Mass Index, *WBC (T*_*0*_*)* white blood cells count at baseline (T_0_), *WBC (T*_*END*_*)* WBCs count at the end of treatment (T_END_), *Δ WBC* differential count of WBCs (T_END_ − T_0_), *Neutr. (T*_*0*_*)* neutrophils count at baseline (T_0_), *Neutr. (T*_*END*_*)* neutrophils count at the end of treatment (T_END_), *Δ Neutr.* differential count of neutrophils (T_END_ − T_0_), *Mono. (T*_*0*_*)* monocytes count at baseline (T_0_), *Mono. (T*_*END*_*)* monocytes count at the end of treatment (T_END_), *Δ Mono.* differential count of monocytes (T_END_ − T_0_), *Lymph. (T*_*0*_*)* lymphocytes count at baseline (T_0_), *Lymph. (T*_*END*_*)* lymphocytes count at the end of treatment (T_END_), *Δ Lymph.* differential count of lymphocytes (T_END_ − T_0_).

**Figure 2:**
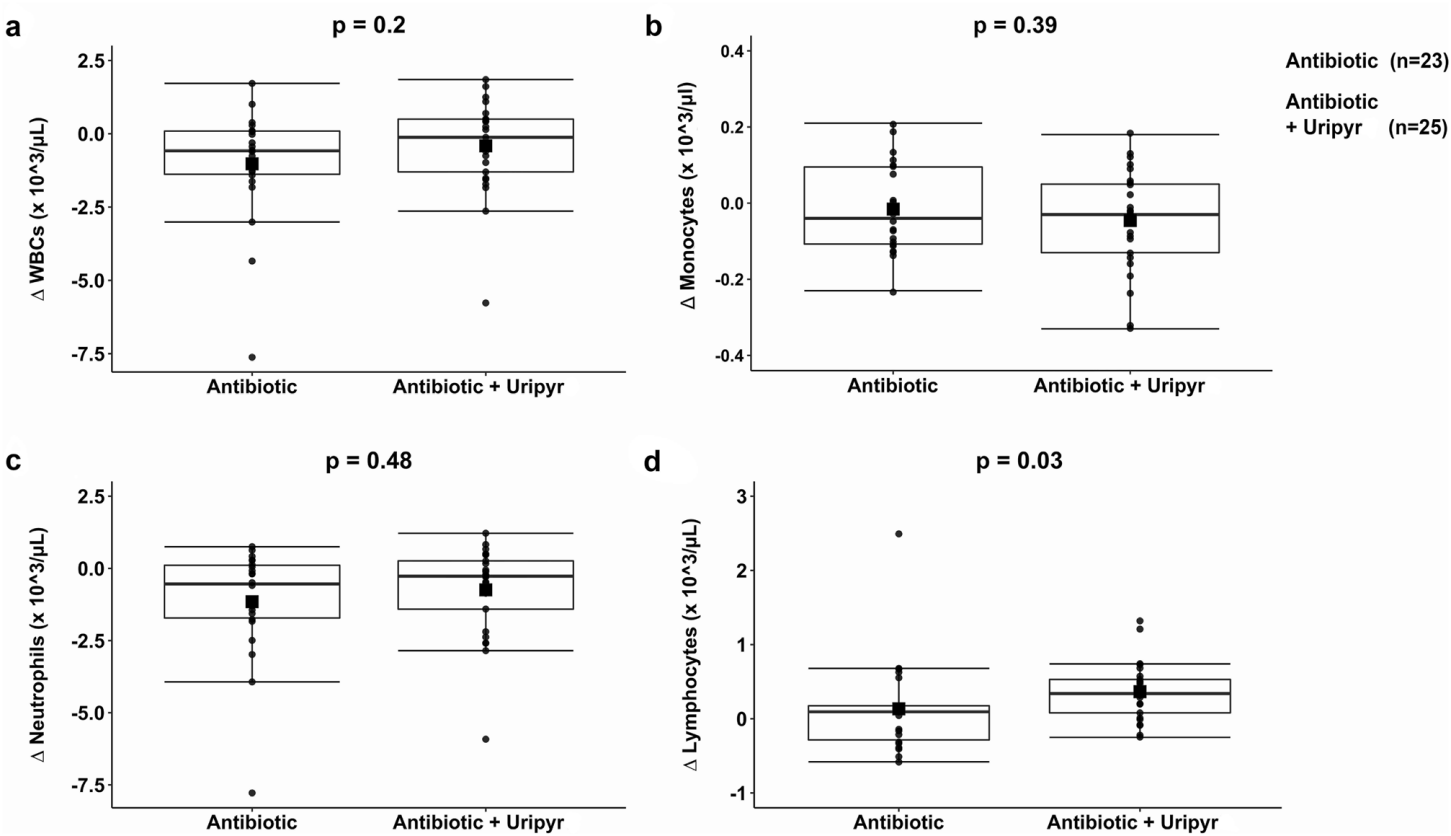
Modulation of blood count by Uripyr oral supplementation in mitotoxic antibiotic-treated patients. Differential value of White Blood Cells (WBCs, **a**), monocytes (**b**), neutrophils (**c**) and lymphocytes (**d**) counts, calculated as delta value between the end of the antibiotic treatment (T_END_) and the baseline (T_0_) in the Uripyr and the control group. Data are expressed as median and [IQR] for a and d and mean ± SD for (**b**) and (**c**). *p < 0.05 Plots and graphs were drawn up using the R^38^.

We firstly investigated if oral supplementation with Uripyr (Mitobiotix, Italy) during antibiotic therapy can induce an in vivo effect. For this reason, we calculated the differential blood cell count in 48 patients between the end of the antibiotic treatment (T_END_) and the baseline (T_0_). Our data showed no significant difference in the White Blood Cells (WBCs) count when comparing the Uripyr group with the control group (Fig. 2a). A similar trend was found for the monocytes (t = − 0.87, df = 45.79) and neutrophils (t = 0.70, df = 43) counts (Fig. 2b,c, respectively). By contrast, a 3.4-fold significant difference was found in the lymphocytes count showing a delta value of 0.34 [0.45] in the Uripyr group versus 0.10 [0.52] in the control group (Fig. 2d, p < 0.05). This relatively small effect in the differential lymphocyte count, while being indicative for a possible protection by uridine and pyruvate supplementation on the lymphocyte survival/replication during the basic therapy with a single mitotoxic antibiotic, can acquire a more significant clinical impact in terms of immune response in the case of long-term and/or combinatorial antibiotic therapies.

### Rescue of proliferative capacity of T cells by Uripyr supplementation in patients treated with mitotoxic antibiotics

To better understand the modulation by Uripyr oral supplementation of the antibiotic-dependent effects on immune cells, we performed an ex vivo proliferation test on cultured PBMCs from 22 patients. Lymphocyte proliferation assays are largely used in clinical diagnosis for monitoring the immune function efficiency^20^. In this assay, ex vivo cultured lymphocytes are challenged with cell-specific activation and expansion *stimulus*. Specifically, our ex vivo study was performed in a new experimental setting in which PBMCs isolated from Uripyr and control group at the end of the antibiotic treatment (T_END_) were cultured in autologous serum, thus avoiding the exogenous supplementation of essential microelements and metabolites normally done with laboratory culture media as well as maintaining blood cells in their specific physiological environment. Since the proliferative *stimulus* utilized in our assay is specific for T cells, we analyzed the cytofluorimetric profile of CD3^+^ cell populations in 12 patients from the Uripyr group and 10 patients from the control group. Cytofluorimetric analysis of CD3^+^ cells gated from total PBMCs showed a 3.7-fold increase in the percentage of dividing cells in the Uripyr group (39.71 ± 4.93) as compared to the control group (10.84 ± 2.77) (Fig. 3, p ≤ 0.0001). This result demonstrates that Uripyr supplementation sustains and protects the T cell proliferative capacity during therapies with mitotoxic antibiotics.

**Figure ﻿3:**
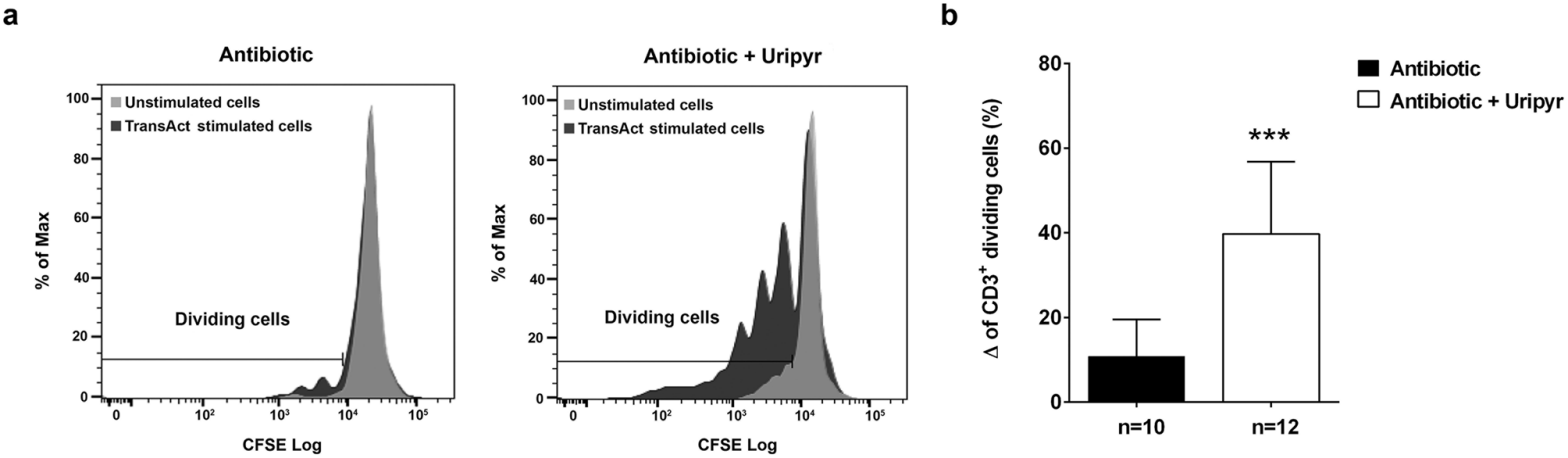
Effects of Uripyr oral supplementation on the ex vivo proliferation of CD3^+^ gated cells from PBMCs of mitotoxic antibiotic-treated patients. (**a**) Representative overlay histograms for CFSE staining of CD3^+^ gated cells from PBMCs of Uripyr and control group. The histograms indicate the proliferating ability of unstimulated (grey histograms) and TransAct stimulated (black histograms) cells. (**b**) The mean percentage of dividing CD3^+^ gated cells from PBMCs was calculated as delta value of TransAct stimulated vs. unstimulated cells after antibiotic treatment—with and without Uripyr supplement (white and black bars, respectively). Data are expressed as mean ± SEM. ***p ≤ 0.0001.

## Discussion

Antibiotics are the first-line therapy for the treatment and prevention of bacterial infections, but they can have multiple off-target actions on human cells causing a wide range of adverse effects^2^. While antibiotics are well tolerated by most people, some side effects can become very serious or even lethal, depending on physiological (e.g. age, pregnancy) and/or specific pathological conditions (e.g. immunodepression, comorbidities, malnutrition). In the case of commonly used antibiotics, the large numbers of people suffering short-term or chronic adverse effects have drawn attention back to drug safety concerns, in some cases leading to an official warning being issued for a very restricted therapeutic usage, as in the recent case of quinolone antibiotics^21^.

In the present paper, we have focused our attention on the effects of mitotoxic antibiotics on immune cells. Due to their off-target inhibition of mitochondrial protein synthesis and DNA replication and transcription, mitotoxic antibiotics can cause actively dividing cells to become dependent on uridine and pyruvate supplementation for their growth, as in the case of mtDNA–depleted (rho0) cells. In this context, we carried out a clinical pilot study in 55 patients with ABU and positive sperm cultures undergoing treatment with mitotoxic antibiotics, with or without uridine + pyruvate (Uripyr, Mitobiotix, Italy) supplementation, to avoid the risk of urinary sepsis, according to EAU guidelines^17^. Our data, obtained both in vivo and by a modified cell proliferation assay of isolated PBMCs cultured ex vivo in autologous serum, show a striking protection of the T cell proliferative capacity in the Uripyr supplementation group.

Mitochondria are currently taking a center stage in immunobiology, not only in terms of bioenergetic functions but also as metabolic and signaling hubs of immune cells^22,23^. In particular, upon antigen recognition, T cells undergo a rapid clonal expansion and differentiate into specific effector subsets whose functions are counteracted by regulatory T cells (Treg). After these processes, only long-living memory T cells survive and can promote a faster and stronger response to a secondary challenge of the same nature^24^. Therefore, mitochondria may be seen as master metabolic regulators of T lymphocytes thanks to their ability to modulate different stages of T cell adaptive responses including migration, activation, proliferation, differentiation, memory phase, and exhaustion^25^. Naïve T cells predominantly use OXPHOS as the principal source of energy, while activated T cells exhibit higher glycolysis. During the T cells differentiation process, a shift towards aerobic glycolysis induces the generation of proinflammatory T cell subsets (Th1 and Th17), while the promotion of OXPHOS leads to the onset of a regulatory phenotype for T cells (Treg and memory T cells)^26^.

In this context, clinical and experimental data have recently demonstrated that the immune system and, specifically, T cells require a functional mitochondrial respiratory chain^27^. Firstly, a retrospective analysis showing recurrent or severe infections in a cohort of pediatric patients with mitochondrial diseases prompted the authors to characterize their T cell populations. The work revealed a baseline paucity of memory T cells as well as leukopenia provoked by episodes of acute infections. Then, they created a mouse model with a T cell-specific knockout of the *COX10* gene (*TCox10*^*−/−*^) encoding an essential assembly factor for cytochrome c oxidase, i.e. the terminal enzyme of the mitochondrial respiratory chain. The *TCox10*^*−/−*^ mice displayed severe abnormalities in T cell activation, differentiation and function as well as an impaired immune response to vaccines and viral infections^27^. In line with the above findings, since the three catalytic subunits of cytochrome c oxidase are encoded by mtDNA, mitotoxic antibiotics by lowering the mtDNA copy number (as in the case of fluoroquinolones), or by inhibiting the mitochondrial protein synthesis, would have a detrimental impact on T cells, as we did indeed observe in our work. It would be interesting to test the effect of uridine and pyruvate supplementation on both the phenotype of *TCox10*^*−/−*^ mice and on the basic properties of the immune system in patients affected by mitochondrial disorders.

Greater adverse effects by mitotoxic antibiotics could occur both in individuals with reduced mitochondrial capacity and/or with primary or acquired immunodeficiencies. In particular, the decay of mitochondrial function and immunosenescence are co-existing and possibly related hallmarks of aging^28^. For this reason, adjuvant supplementation with uridine and pyruvate would also be desirable when treating elderly people with mitotoxic antibiotics and/or to boost their immune system for a better response to vaccines^29^.

Besides its classical role in RNA and DNA synthesis, uridine can have multi-targeted effects due to its conversion or incorporation in other molecules with different biological actions^30^. Therefore, the inhibition of endogenous uridine biosynthesis by mitotoxic antibiotics can be associated with other tissue-specific adverse effects. For instance, uridine triphosphate (UTP) is used to activate glucose-1P to UDP-glucose, then oxidized to UDP-glucuronate, required for the biosynthesis of glycosaminoglycans, such as heparan sulfate, chondroitin sulfate and hyaluronic acid. These essential components of the extracellular matrix interact with collagens and other proteins, thus playing an essential role in connective tissue function. In this light, the increased risk associated to the use of fluoroquinolones^31^, of collagen-related adverse effects such as tendinitis and tendon rupture, aortic aneurysm or aortic dissection, might also be prevented by uridine and pyruvate supplementation during long-term or recurrent therapies with this group of antibiotics.

Moreover, protecting the immune system during mitotoxic antibiotic therapies could be of the utmost importance to reduce the onset of antibiotic resistance. In fact, during bacterial infections, a synergic action of the antimicrobial activity of the antibiotic together with a first-line defense by cell-mediated immunity towards antibiotic-resistant, persistent or tolerating bacteria, could more efficiently achieve a complete eradication of the pathogen, reducing the risk of recurrent infections.

Our findings might also have important implications in the use of antibiotics in the husbandry of livestock. Adjuvant supplementation with uridine and pyruvate, by boosting the animal immune system especially during metaphylaxis (control) or prophylaxis (prevention) treatments with broad-spectrum and low-cost mitotoxic antibiotics, could help to tackle antimicrobial resistance^32^.

Finally, our findings contribute to optimizing the use of numerous groups of current antibiotics gaining time for the search and the approval of new molecules to enter the clinical pipeline. Together with a better knowledge of the structure of eukaryotic and prokaryotic ribosomes, supporting a deeper understanding of the interactions of antibiotics that target ribosomes and inhibit protein synthesis^33^, this may lead to a re-evaluation of old molecules and an improvement of their pharmacological effects, also making them more bacterial target-specific thus further reducing their toxicity.

In conclusion, this work shows that adjuvant supplementation with uridine and pyruvate protects the proliferative capacity of T lymphocytes, effector cells of cell-mediated immunity, from mitotoxic antibiotics. Our results lay the groundwork for a “renaissance” of forgotten antibiotic molecules and for improving antibiotic medicine in terms of anti-microbial efficacy, administration protocols, and clinical application potentials. This may be an important step forward in the antibiotic resistance era.

## Methods

### Study participants

A population of subjects scheduled to undergo endourological maneuvers, prostate biopsy, or medically-assisted reproduction was selected in the Urology, Andrology, and Kidney Transplantation Units of the University of Bari. Among them, we enrolled those with asymptomatic bacteriuria or with positive sperm culture requiring antibiotic treatment according to EAU guidelines^17–19,34,35^. The population did not include patients with acute and/or life-threatening diseases like a renal and hepatic failure, nor patients with infections, acute stress, or those in treatment with medications affecting the number of leukocytes (i.e. corticosteroids). Other exclusion criteria were: infections sustained by Multi-Drug Resistant (MDR), Xtreme Drug-Resistant (XDR), Pan Drug-Resistant (PDR) microbes; chronic systemic inflammatory diseases; neoplastic diseases of recent onset (less than 10 years) and/or under chemotherapeutic treatment; immune-depression conditions; pregnant and/or puerperal women; allergy and/or adverse reactions to the prescribed drug or its excipient. The study was conducted in compliance with the Helsinki Declaration and was approved and registered (registration number: 5251) by the Ethical Committee of the Azienda Ospedaliero-Universitaria Policlinico di Bari, Italy, officially accredited to the Italian Medicines Agency (AIFA). Between July 2017 and December 2019, 59-sequential eligible patients were recruited and after obtaining informed consent, 55 were finally enrolled in the trial. All the selected subjects belonged to Caucasian ethnicity and were predominantly men (85.18% of controls and 75% of the group treated with Uripyr, Mitobiotix, Italy). The mean age of patients of the Uripyr group was 58.32 ± 16.12 years vs. the 53.78 ± 16.73 of controls, meanwhile the BMI was similar in the two groups (25.46 ± 2.86 Uripyr group vs. 25.81 ± 3.30 of controls). The percentages of chronic comorbidities and nephrological diseases did not differ considerably in the two experimental groups. The primary endpoint of the present study was the analysis of the effects of oral supplementation with Uripyr during treatment with mitotoxic antibiotics on hemogram evaluations; the second endpoint was the *exvivo* investigation of immune cell functional properties within the same cohort of patients.

### Hemogram analysis

The enrolled patients underwent a complete blood count, urinalysis, and urine and/or semen culture (according to the clinical judgment) at baseline (T_0_), before starting any antibiotic treatment. Demographic and clinical data were also collected at this time. Then, participants were randomly allocated to the “Antibiotic treatment plus Uripyr” (Uripyr) group or “Antibiotic treatment without Uripyr” (control) group. The randomization process and subsequent assignment to the intervention group were managed by an independent pharmacological consultant. Figure 1 shows a flow diagram of the population selection process. Patients in the Uripyr group were instructed to take three doses of Uripyr (each containing 150 mg of uridine and 2 g of pyruvate) per day, one hour before taking the antibiotic. Each subject was treated with a single antibiotic according to the culture test susceptibility results and the duration and dosage described by the best-accepted guidelines^17,18,35^. The mitotoxic antibiotics were administered at the following doses: levofloxacin 500 mg qd (7–10 days), ciprofloxacin 500 mg bid (7–10 days), tigecycline 50 mg bid (for 7 days), doxycycline 100 mg bid for the first day and then qd (7–10 days), linezolid 600 mg bid (10 days), erythromycin 1 g bid (10 days). Finally, at the end of the antibiotic therapy, each patient repeated the same clinical evaluations as at baseline (defined as T_END_ values). In addition, proinflammatory markers such as CRP (C-reactive protein) were available in most of the patients. Adverse effects, if any, were recorded. The use of any other phytotherapeutic agents for genital and urinary symptoms (e.g. Serenoa repens^36^) was also checked.

### Proliferation test of CD3^+^ cells

Peripheral blood mononuclear cells (PBMCs) were freshly isolated from venous blood drawn into BD Vacutainer Heparin Tubes using Ficoll Paque Plus (GE Healthcare, USA) according to the manufacturer’s instructions. PBMCs were collected after the antibiotic treatment (T_END_) with or without the Uripyr supplementation. PBMCs isolated from each patient were washed in sterile PBS 1X (Euroclone) and stained with CellTrace CFSE (Thermo Fisher Scientific, MA, USA) for 5 min at 37 °C (1 μL/ml PBS1X). CFSE staining was stopped by diluting cells with five volumes of BSA1%-PBS1X (Bovine Serum Albumin, Sigma-Aldrich, USA) for 5 min at room temperature. Stained PBMCs from each patient were washed again in PBS1X and plated in 100% of autologous heat-inactivated serum for 30 min at 37 °C, adjusting cell density to 1 × 10^6^ cells/ml in 48 wells. After seeding, cells were stimulated with 10 μL of TransAct (Miltenyi Biotec, Bergisch Gladbach, Germany) according to the manufacturer’s instructions and maintained at 37 °C in a humidified atmosphere of CO_2_/air (5:95). After 72–96 h of culture, cells were collected, washed in PBS1X, and stained for 20 min with 1:20 diluted anti-CD3 antibody (CD3-PE-Vio 770, human, REA613 Clone—Miltenyi Biotec, Bergisch Gladbach, Germany). Cytofluorimetric analysis was carried out in a blind setup by two different groups using the FC500 Flow cytometer (Beckman Coulter, CA, USA) and the FACSCanto II (Becton, Dickinson and Company, OR, USA), respectively. Data analysis was performed using FlowJo software, version 10.6.1 (Becton, Dickinson and Company, OR, USA). Gating strategy: forward scatter vs*.* side scatter for PBMCs gating. Gated PBMCs were checked for positivity to CD3 (forward scatter vs. CD3). CD3^+^ gated cells were analyzed for CFSE staining by histogram to test their proliferating ability. The percentage of dividing cells from each population was calculated as a delta of the mean percentages of TransAct stimulated versus unstimulated cells (antibiotic treatment ± Uripyr).

### Statistical methods

The clinical study was conducted with a 1:1 randomization according to the extension of the CONSORT Statement to randomized plot and feasibility trials^37^. Statistical analyses were performed using the R statistical environment, packages “stats”, “car”, “gmodels”, “fBasics” and “pwr”. We performed a sample size calculation with a medium (0.5) and large (0.8) effect size, 80% power and two-sided 5% significance, yielding trial sample sizes of 64 and 26 subjects for each treatment arm, respectively. These evaluations may be useful for a future randomized control trial. Shapiro–Wilk tests and graphic evaluations of each variable were performed to test for normal distribution. All the quantitative variables were considered as normally distributed, except for differential values of white blood cells and lymphocytes. Descriptive statistics are presented as mean $\pm$ standard deviation and/or median and [IQR], for normally distributed and non-normally distributed continuous variables, respectively, whereas categorical variables are indicated as frequency (%). Then, two-sided Student t-test and Mann–Whitney’s U test, as appropriate, were performed to assess comparisons among the two groups. Differences in categorical variables between groups were assessed by the Pearson χ^2^ test. Plots and graphs were drawn up using the R package “graph” and “ggplot2” for the in vivo study and the GraphPad Prism statistical software release 6.01 for the ex vivo study. For the ex vivo analysis, the unpaired student t-test was used; data are expressed as mean ± SEM. A level of significance of p < 0.05 was used to compare the two groups.

## Supplementary Information

Supplementary Information.
