# Supplementary material for: Uridine and pyruvate protect T cells’ proliferative capacity from mitochondrial toxic antibiotics: a clinical pilot study
Source: Sci Rep. 2021 Jun 18;11:12841. doi: 10.1038/s41598-021-91559-8 (PMC8213784; doi:10.1038/s41598-021-91559-8)
Supplement: Supplementary file 1 — Supplementary Information. [file 41598_2021_91559_MOESM1_ESM.pdf]

Uridine and pyruvate protect T cells' proliferative capacity from mitochondrial toxic antibiotics: a clinical pilot study

Stefano Battaglia[1,2§], Stefania De Santis[1,3§], Monica Rutigliano[2], Fabio Sallustio[1], Angela Picerno[2], Maria Antonia Frassanito[4], Ingo Schaefer[5], Angelo Vacca[4], Antonio Moschetta[1], Peter Seibel[5], Michele Battaglia[2]* and Gaetano Villani[6]*.

[§] These authors contributed equally to the work

* Corresponding authors

[1] Department of Interdisciplinary Medicine, "Aldo Moro" University of Bari, 70124 Bari, Italy;
[2] Department of Emergency and Organ Transplants, "Aldo Moro" University of Bari, 70124 Bari, Italy;
[3] Department of Pharmacy-Drug Science, "Aldo Moro" University of Bari, 70126 Bari, Italy;
[4] Department of Biomedical Sciences and Human Oncology, "Aldo Moro" University of Bari, 70124 Bari, Italy;
[5] Molecular Cell Therapy, BBZ, Medical Faculty, University of Leipzig, 04103 Leipzig, Germany;
[6] Department of Basic Medical Sciences, Neuroscience and Sense Organs, "Aldo Moro" University of Bari, 70124 Bari, Italy.

* To whom correspondence should be addressed:
E-mail: michele.battaglia@uniba.it; gaetano.villani@uniba.it

Supplementary Tables

Table S1. Demographic and clinical features of the enrolled patients with hemogram evaluation.

Variable	Uripyr group	Control group	NA
**Number**	**25**	**23**	**-**
**Age** (years)*	60.68±15.07	53.91±16.33	
**BMI** (kg/m^2)*	25.61±2.93	25.87±3.39	5
**WBC (T_0)** (x10^3 /μL)**	6.50 [1.89]	6.27 [1.87]	
**WBC (T_{END})** (x10^3 /μL)**	6.44 [1.58]	5.51 [1.58]	
**Δ WBC** (x10^3 /μL)**	-0. 12 [1.8]	-0.56 [1.44]	
**neutr. count (T_0)** (x10^3/μL)*	4.20±1.67	4.03±2.13	
**neutr. count (T_{END})** (x10^3 /μL)*	3.46±0.95	2.93±0.83	
**Δ neutr.** (x10^3 /μL)*	-0.74±1.58	-1.10±1.90	
**mono. count (T_0)** (x10^3/μL)*	0.50±0.20	0.44±0.14	
**mono. count (T_{END})** (x10^3 /μL)*	0.45±0.15	0.42±0.20	
**Δ mono.** (x10^3 /μL)*	-0.05±0.13	-0.01± 0.12	
**lymph. count (T_0)** (x10^3 /μL)**	2.05 [0.52]	1.87 [0.81]	
**lymph. count (T_{END})** (x10^3 /μL)**	2. 38 [0.91]	1.90 [0.84]	
**Δ lymph.** (x10^3 /μL)**	0.34 [0.45]	0.10 [0.52]	
**Sex**			
**Male**	18 (72.00)	20 (86.95)	
**Female**	7 (28.00)	3 (13.04)	
**Smoke**			
**Non smokers**	22 (88.00)	19 (86.36)	1
**Smokers**	3 (12.00)	3 (13.63)	
**General comorbidity**			

**Absence**	13 (52.00)	14 (60.86)	
**Presence**	12 (48.00)	9 (39.13)	
**Nephrological disease**			
**Absence**	23 (92.00)	22 (95.65)	
**Presence**	2 (8.00)	1 (4.34)	
**Nephrolithiasis**			
**Absence**	23 (92.00)	22 (95.65)	
**Presence**	2 (8.00)	1 (4.34)	
**Germ location**			
**Urine**	18 (72.00)	8 (34.78)	
**Sperm**	7 (28.00)	15 (65.22)	
**Antibiotic treatment**			
**Quinolones**	17 (68.00)	13 (56.52)	
**Macrolides**	0 (00.00)	4 (17.39)	
**Tetracyclines**	8 (32.00)	5 (21.73)	
**Oxazolidinones**	0 (00.00)	1 (4.34)	

Values are expressed as mean ± Standard Deviation or median [IQR] respectively for normal (*) and non-normal (**) distributed numeric variables, and with n (%) for categorical ones. Each item was compared among the 2 groups using t-test or Mann-Whitney's U test for quantitative variable and Pearson χ^2 test for categorical ones. A level of significance of $P< 0.05$ (two-sided) was used to compare Uripyr and control group. **Abbreviations: NA,** Not Available**; BMI,** Body Mass Index; **WBC (T_0)**, White Blood Cells count at baseline (T_0); **WBC (T_{END})**, WBCs count at the end of treatment (T_{END}); **Δ WBC**, differential count of WBCs (T_{END} - T_0); **neutr. (T_0)**, neutrophils count at baseline (T_0); **neutr. (T_{END})**, neutrophils count at the end of treatment (T_{END}); **Δ neutr.**, differential count of neutrophils (T_{END} - T_0); **mono. (T_0)**, monocytes count at baseline (T_0); **mono. (T_{END})**, monocytes count at the end of treatment (T_{END}); **Δ mono.**, differential count of monocytes (T_{END} - T_0); **lymph. (T_0)**, lymphocytes count at baseline (T_0); **lymph. (T_{END})**, lymphocytes count at the end of treatment (T_{END}); **Δ lymph.**, differential count of lymphocytes (T_{END} - T_0).

Table S2. Demographic and clinical features of the enrolled patients with PBMCs evaluation.

Variable	Uripyr group	Control group	NA
**Number**	**12**	**10**	**-**
**Age** (years)*	62.08±13.83	57.20±15.65	
**BMI** (kg/m^2)*	26.47±3.11	25.54±2.29	3
**WBC (T_0)** (x10^3 /μL)**	5.80 [1.68]	6.01 [2.64]	1
**WBC (T_{END})** (x10^3 /μL)**	6.50 [2.41]	5.56 [2.19]	2
**Δ WBC** (x10^3 /μL)**	0.42 [0.96]	-0.02 [1.32]	1
**neutr. count (T_0)** (x10^3 /μL)*	3.73±1.30	3.46±1.71	1
**neutr. count (T_{END})** (x10^3 /μL)*	3.58±1.30	2.82±1.00	2
**Δ neutr.** (x10^3 /μL)*	-0.15±0.99	-0.92±1.94	1
**mono. count (T_0)** (x10^3 /μL)*	0.46±0.21	0.46±0.14	1
**mono. count (T_{END})** (x10^3/μL)*	0.46±0.15	0.49±0.23	2
**Δ mono.** (x10^3 /μL)*	0.00±0.13	-0.02± 0.18	1
**lymph. count (T_0)** (x10^3 /μL)**	2.09 [0.67]	1.58 [0.53]	1
**lymph. count (T_{END})** (x10^3 /μL)**	2.48 [0.79]	1.88 [0.57]	2
**Δ lymph.** (x10^3 /μL)**	0.29 [0.75]	0.10 [0.49]	1
**Sex**			
**Male**	9 (86.36)	10 (100.00)	
**Female**	3 (13.63)	0 (00.00)	
**Smoke**			
**Non smokers**	11 (91.66)	9 (90.00)	
**Smokers**	1 (8.33)	1 (10.00)	
**General comorbidity**			

**Absence**	5 (41.66)	6 (60.00)	
**Presence**	7 (58.33)	4 (40.00)	
**Nephrological disease**			
**Absence**	11 (91.66)	9 (90.00)	
**Presence**	1 (8.33)	1 (10.00)	
**Nephrolithiasis**			
**Absence**	12 (100.00)	10 (100.00)	
**Presence**	0 (0.00)	0 (0.00)	
**Germ location**			
**Urine**	8 (66.66)	4 (40.00)	
**Sperm**	4 (33.32)	6 (60.00)	
**Antibiotic treatment**			
**Quinolones**	7 (58.33)	7 (70.00)	
**Macrolides**	0 (00.00)	0 (00.00)	
**Tetracyclines**	5 (41.66)	3 (30.00)	
**Oxazolidinones**	0 (00.00)	0 (00.00)	

Values are expressed as mean ± Standard Deviation or median [IQR] respectively for normal (*) and non-normal (**) distributed numeric variables, and with n (%) for categorical ones. Each item was compared among the 2 groups using t-test or Mann-Whitney's U test for quantitative variable and Pearson χ^2 test for categorical ones. A level of significance of $P< 0.05$ (two-sided) was used to compare Uripyr and control group. **Abbreviations: NA,** Not Available**; BMI,** Body Mass Index; **WBC (T_0)**, White Blood Cells count at baseline (T_0); **WBC (T_{END})**, WBCs count at the end of treatment (T_{END}); **Δ WBC**, differential count of WBCs (T_{END} - T_0); **neutr. (T_0)**, neutrophils count at baseline (T_0); **neutr. (T_{END})**, neutrophils count at the end of treatment (T_{END}); **Δ neutr.**, differential count of neutrophils (T_{END} - T_0); **mono. (T_0)**, monocytes count at baseline (T_0); **mono. (T_{END})**, monocytes count at the end of treatment (T_{END}); **Δ mono.**, differential count of monocytes (T_{END} - T_0); **lymph. (T_0)**, lymphocytes count at baseline (T_0); **lymph. (T_{END})**, lymphocytes count at the end of treatment (T_{END}); **Δ lymph.**, differential count of lymphocytes (T_{END} - T_0).
